# Sulphur‐doped carbon dots as a highly efficient nano‐photodynamic agent against oral squamous cell carcinoma

**DOI:** 10.1111/cpr.12786

**Published:** 2020-04-16

**Authors:** Qirong Li, Ronghui Zhou, Yu Xie, Yanjing Li, Yu Chen, Xiaoxiao Cai

**Affiliations:** ^1^ State Key Laboratory of Oral Diseases National Clinical Research Center for Oral Diseases West China Hospital of Stomatology Sichuan University Chengdu China; ^2^ West China School of Stomatology, Oral pathology Sichuan University Chengdu China

**Keywords:** apoptosis, nano‐photodynamic agent, oral squamous cell carcinoma, sulphur‐doped carbon dots

## Abstract

**Objectives:**

Photodynamic therapy (PDT) is a novel non‐invasive therapeutic method, which has been widely applied for the treatment of human oral cancers. However, the problems of undesirable singlet oxygen (^1^O_2_) quantum yields and long‐term phototoxicity were inevitable during the application of traditional photosensitizers. Therefore, it is necessary to explore novel photosensitizers for the improvement of therapeutic effects. In our study, the sulphur‐doped carbon dots (S‐CDs) of high yield of singlet oxygen (^1^O_2_) were synthesized as a nano‐photosensitizer for OSCC to improve the PDT efficacy in clinical practice.

**Materials and methods:**

After synthesis of the novel S‐CDs, the size, morphologic characteristics, surface potential and yield of singlet oxygen (^1^O_2_) were determined. In vitro study was performed to compare the therapeutic effect as well as the biocompatibility of the novel S‐CDs to those of 5‐ALA. Besides, possible mechanism of action was illustrated.

**Results:**

After synthesis of the novel S‐CDs, the size, morphologic characteristics, surface potential and yield of singlet oxygen (^1^O_2_) were determined. In vitro study was performed to compare the therapeutic effect as well as the biocompatibility of the novel S‐CDs to those of 5‐ALA. Besides, possible mechanism of action was illustrated.

**Conclusions:**

These data from the in vitro study demonstrated the promising safety profile of the low dose (nmol/L) S‐CDs, which indicated the novel S‐CDs could be used as a promising photodynamic agent for oral cancer therapy.

## 1 INTRODUCTION

Squamous cancer is a common malignant oral carcinoma located in the epidermal or adnexal cells. To date, the treatment methods for squamous cell carcinoma mainly includes surgery, freezing and radiotherapy, which can either directly remove or destroy tumour tissues. However, due to the non‐negligible side effects, large wound and potential psychological disorders, the application of the above conventional methods could be limited. Recently, a simple non‐invasive and simple therapeutic strategy, namely photodynamic therapy (PDT), has been widely applied in clinical cancer treatments.^1^, ^2^ Generally, the toxic effect of the reactive oxygen species (ROS) generated by transferring energy from light to oxygen molecules can directly kill tumour cells through photosensitizers.^3^ Besides, ROS can damnify the vasculature of tumour parenchyma, resulting in blood supply deficiency,^4^, ^5^ and further provoke the anti‐tumour immune response further.^3^, ^6^ What is more, even repeated use of PDT can achieve satisfactory therapeutic outcome without causing obvious side effects or any scar formation.^7^ Due to the superficial nature of oral and skin tumours, tumour tissue is located in an area that is easy to receive light, which facilitates the efficient implementation of PDT.^7^ Therefore, PDT has been regarded as an alternative or supplementary therapeutic method of the traditional regimens for the treatment of superficial tumours.

Typically, PDT is a three‐way process consisting of photosensitizer (PS), light and molecular oxygen.^8^ The concentration of ROS directly affects the efficiency of PDT, which largely relies on the photo‐oxidation ability of the PSs.^9^ Over the last few decades, photosensitizers in clinical use are the first and second generation of organic PSs have been approved for clinical use, such as haematoporphyrin (Hp), photofrin and chlorin e6 (Ce6), among which 5‐aminolevulinic acid (ALA)‐mediated PDT has been widely applied in clinical practice with satisfactory outcomes.^8^, ^10^, ^11^ 5‐ALA is the biological precursor of protoporphyrin IX (PpIX). Several previous studies^12^, ^13^, ^14^ have demonstrated its superiority in the treatment of both oral pre‐cancer and oral cancer treatment since it induces the accumulate high concentration of PpIX in cancer cells. However, certain deficiencies, such as 5‐ALA suffered from long‐term phototoxicity and poor cell penetration, would lower the PDT therapeutic effect, thus limiting the clinical application of 5‐ALA. Therefore, the third generation of photosensitizer came into being to solve the problems of the first two generations. Wang and his co‐workers^15^ recently developed a nanosystem by combining chemotherapy with PDT for the targeted treatment of OTSCC, and they found that CAL‐27 cells assimilated PEG linked haematoporphyrin (HP) and depicted high PDT efficiency through the target‐peptide‐mediated cell internalization. Although the PDT efficiency has been assembly promoted by using biomolecular modification and nano‐materials technologies, certain problems, such as poor biocompatibility, weak selectivity and fluorescence quenching under light,^16^ are still inevitable.

**FIGURE 1:**
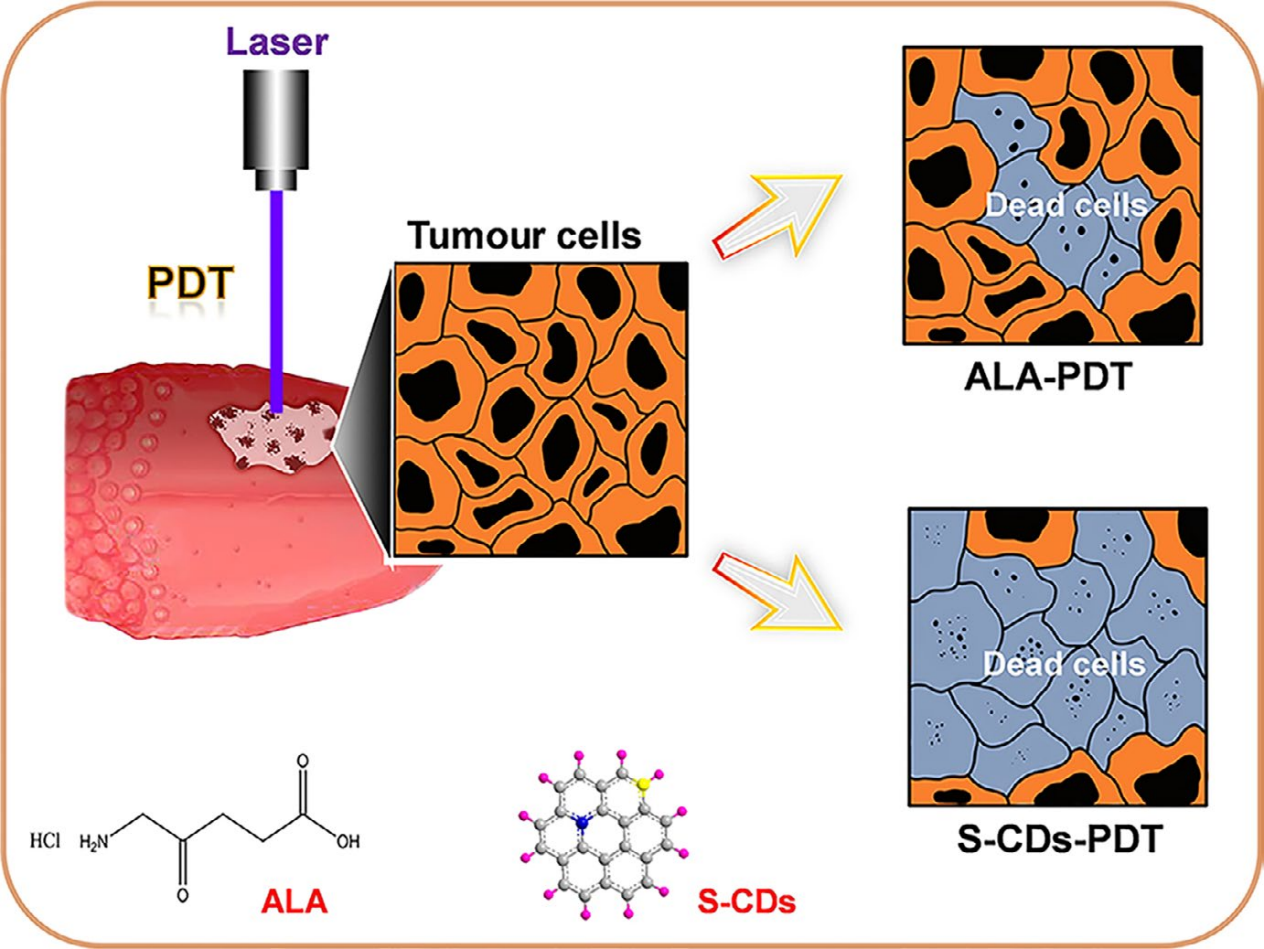
Illustration for the S‐CDs and 5‐ALA‐mediated PDT in UM1 cells

As the fourth generation of PSs, nano‐photosensitive agents possess excellent characteristics of adjustable excitation and emission wavelength, strong anti‐photobleaching ability, and good biocompatibility.^17^ Carbon‐based nano‐materials, such as carbon nanotubes,^18^, ^19^ graphene^20^ and carbon dots,^21^, ^22^, ^23^ have been drawing more and more attention in the fields of cancer diagnosis and therapy. In this study, the sulphur‐doped carbon dots (S‐CDs)^24^ with a high single oxygen quantum yield were prepared for the PDT treatment for oral squamous cell carcinoma. The S‐CDs could automatically enter cancer cells and instigate potent trigger cancer cell death by light. Moreover, by detecting the expression of apoptotic proteins, we also observed that the UM1 cells treated with S‐CDs had higher expression levels of apoptotic proteins compared with those treated with classic photosensitizer 5‐ALA at the same concentration (Figure 1), indicating the promising application prospect of the developed nano‐photosensitizer in the PDT treatment for oral cancer treatment.

## 2 MATERIALS AND METHODS

### 2.1 Preparation and characterization of S‐CDs

Sulfur‐doped carbon dots were prepared via a hydrothermal process using polythiophene (PT2) as the precursor that developed by Wang et al^24^ The detailed synthesis steps are as follows: PT2 (30 mg) and NaOH solution (40 mL, 0.5 mmol/L) were firstly mixed for ultrasound for 30 minutes, and then transferred the mixture into an autoclave and maintained the temperature at 170°C. After reaction for 24 hours, the product were purified with 0.22‐µm membranes to remove residue and finally dissolved in water.

For characterization, atomic force microscopy (AFM, Shimadzu) and transmission electron microscopy (TEM, JEOL) were applied for investigating the morphology and dimension of S‐CDs, and dynamic light scattering (DLS) was used for studying the size distribution. UV‐Vis and fluorescence spectroscopy of the S‐CDs were measured by UV‐Vis spectrophotometer (Hitachi, U‐3900H) and fluorospectrophotometer (Shimadzu), respectively. Characterization of singlet oxygen of S‐CDs was explored by Fluorolog‐3 spectrofluorometer (Horiba Jobin Yvon).

### 2.2 Cell culture

UM1 cell line originated from a HNSCC‐diagnosed patient.^25^, ^26^ These cell lines were gifts from Dr Chen (Sichuan University). UM1 cells were incubated in DMEM, supplemented with 10% FBS, 100 U/mL penicillin and 100 µg/mL streptomycin (GIBCO). The culture media was renewed twice or three times a week. Cells were put in incubator with temperature at 37°C and 5% CO_2_.

### 2.3 S‐CD uptake and in vitro imaging

Cell suspension was removed, density of 1 × 10^5^/mL, from culture flask and then transferred to 12‐well plates, and 1 µmol/L S‐CDs were added for 0 hour, 3 hours, 6 hours, 12 hours, 24 hours and 48 hours, respectively. The trypsin‐treated UM1 cells were clustered by centrifugation, and then washed with PBS and finally resuspended into 400 µL PBS for flow cytometry test (Attune NxT, Life). In addition, to observe cellular assimilation state and obtain images, confocal laser microscope was employed. Cells, rinsed by PBS, were fixed with 4% cold paraformaldehyde for 15 minutes. After three times' PBS rinsing, phalloidin and DAPI were used to tint the cytoskeleton and nucleus, respectively.

### 2.4 Cellular organelle localization

UM1 cells after incubation with S‐CDs were loaded with MitoTracker green (a mitochondria probe) and LysoTracker green (a lysosome probe), respectively. Samples were washed with PBS after 30 minutes incubation. Analysed photographs were taken from confocal laser scanning microscopy (AIR‐MP, Nikon).

### 2.5 Determine reactive oxygen species (ROS)

The detected green fluorescence, produced by dichlorofluorescein (DCF), was in accordance with intracellular amount of ROS.^27^ Cells grew in 12‐well culture dishes were washed once by PBS and then were maintained at 37°C for 20 minutes with the participation of 2′,7′‐dichlorofluorescein diacetate (DCFH‐DA) (Beyotime). Redo the wash process as previously mentioned and shoot DCFH fluorescence intensity picture of each well on fluorescent inverted microscope (Leica DMRA2; Leica): excitation wavelength was 488 nm; emission wavelength was 530 nm.

Additionally, we performed flow cytometry to record ROS fluorescence signal of each group by cell suspension that had hatched with DCFH‐DA detector. Every group has three accessory holes.

### 2.6 Cell counting Kit‐8 Assay

UM1 cell suspension, at a density of 1 × 10^4^ per well, was cultured in 96‐well plates for 12 hours. Afterwards, S‐CDs and 5‐aminolevulinic acid (ALA) (Sigma‐Aldrich; Vienna, Austria) were added into the medium at concentrations of 5 nmol/L, 10 nmol/L, 50 nmol/L, 100 nmol/L and 500 nmol/L. After 24‐hour incubation, target wells were irradiated with ultraviolet light‐emitting diode (LED) (S‐CDs, *λ* = 420 nm, 2.8125 W, ALA, *λ* = 420 nm, 2.8125 W) and then cultured cells for another 24 hours. After washing with PBS, CCK‐8 solution was put in each tested well to assess the viability of UM1 cells. The optical densities (OD) were measured at 450 nm after 1‐hour incubation at 37°C.

### 2.7 AO/PI test

Cell viability could also be detected through acridine orange‐propidium iodide (AO/PI) kit.^28^ The UM1 cells were cultured in 12‐well plates with the protocol described before (Western blot test). The AO/PI stain procedure briefly is keeping the working solution, containing AO: 670 μmol/L, PI: 750 μmol/L, with cell in the dark at 4°C for 20 minutes and observed each well via fluorescent microscope. Live cells turned into green (AO), whereas the dead appeared red (PI).

### 2.8 Calcium concentration (Ca^2+^) detection

Experimental group and cell treatment were the same as ROS assay. What was slightly different was that the cells were cultured with Fluo‐4/AM (1 µmoL) (Beyotime)^29^ in the incubator for 30 minutes. When entering into cell and meet Ca^2+^, Fluo‐4/AM would transfer to a strongly fluorescent compound. Subsequently, images were captured on fluorescent inverted microscope.

### 2.9 Western blotting

Growth‐arrested UM1 cells were divided into three groups: the control group, ALA (10 nmol/L) with irradiation group and S‐CDs (10 nmol/L) with irradiation group. Then, all groups were harvested 12 hours after the treatment. The whole‐cell lysis assay kit (KeyGen) was used to extract proteins. Protein was denatured in sodium dodecyl sulphate (SDS) buffer at 100°C for 5 minutes, dissociated by 12% SDS‐PAGE afterwards and transferred to PVDF membranes. Then, target proteins were incubated with the correspondent primary antibody Bax, Bcl‐2 and caspase‐3 on the first day and secondary antibody (Beyotime) on the coming day. Subsequently, developer (Bio‐Rad) was added to show the target bands. Band quantification was carried out with the ImageJ software.

### 2.10 Immunofluorescence

To make an entry for antibody to go through the sample cells: fixed by room‐temperatured 4% (w/v) paraformaldehyde for 10 minutes at the punctual time post‐PDT, were punched by room‐temperatured 0.5% Triton X‐100 for additional 10 minutes. Primary antibodies against Bax, Bcl‐2 and caspase‐3 and secondary antibody were applied to form a camera‐detectable fluorescent conjunction afterwards. Then after PBS rinsing, slides were dyed by cytoskeleton and nuclei stainer, and images were captured under confocal laser scanning microscope (AIR‐MP).

### 2.11 Statistical analysis

SPSS 18.0 provided the function of *t* test to tell the differences between each two groups. It is when the *P* value is <.05 that the variant would be considered significant.

## 3 RESULTS

### 3.1 Characterization of the S‐CDs

**FIGURE 2:**
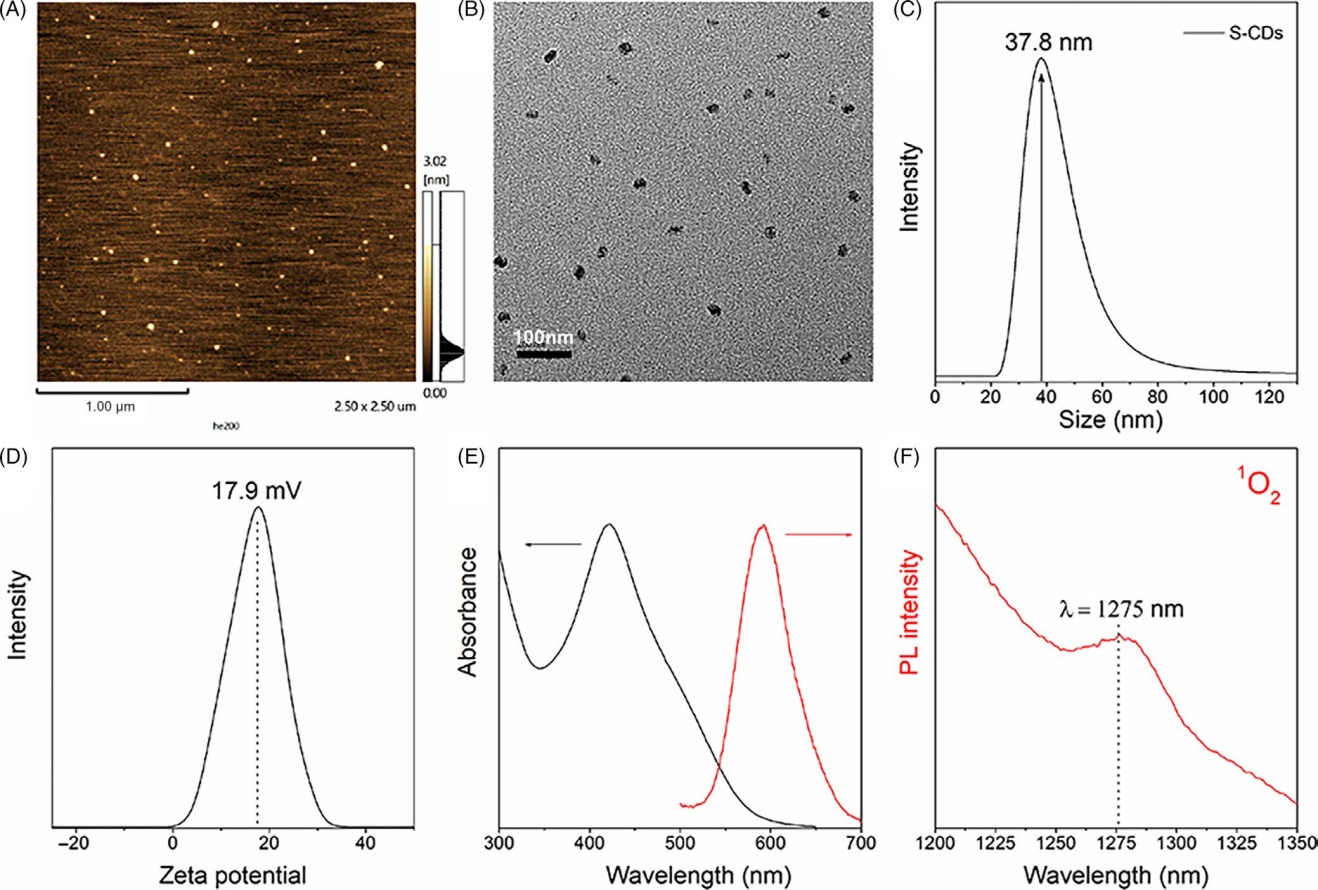
Characterization of S‐CDs: (A) AFM image; (B) TEM image; (C) dynamic light scattering; (D) Zeta potential; (E) UV‐Vis and fluorescent spectroscopy; (F) characterized 1O2 phosphorescence emissions at 1275 nm (CH3CN‐D2O mixture solvent, 15:1, V/V)

The S‐CDs were synthesized as a novel nano‐photosensitizer for oral squamous cell carcinoma based on a polythiophene precursor (PT2). The AFM image displayed a sphere‐like morphology, with a diameter of about 22.26 nm and a height of approximately 4.28 nm (Figure 2A). The particle size of the S‐CDs was determined by the TEM to further study the structure, and the diameter of approximately 28 nm (Figure 2B) was consistent with the average hydrodynamic diameter determined by dynamic light scattering (DLS) (Figure 2C).

Besides, the as‐prepared S‐CD nano‐PS possessed a positive potential of about 17.9 mV (Figure 2D), which is conducive to cellular uptake. In addition, optical characteristic examination (Figure 2E), indicated a broad UV‐Vis absorption ranging from 360 to 600 nm. Meanwhile, an emission band centred at ∼600 nm was obtained, which can be ascribed to the sulphur doping. Furthermore, when the S‐CDs was excited at 420 nm, the characteristic phosphorescence of singlet oxygen at 1275 nm can be observed from S‐CDs (Figure 2F).

### 3.2 Cellular localization of the S‐CDs

**FIGURE 3:**
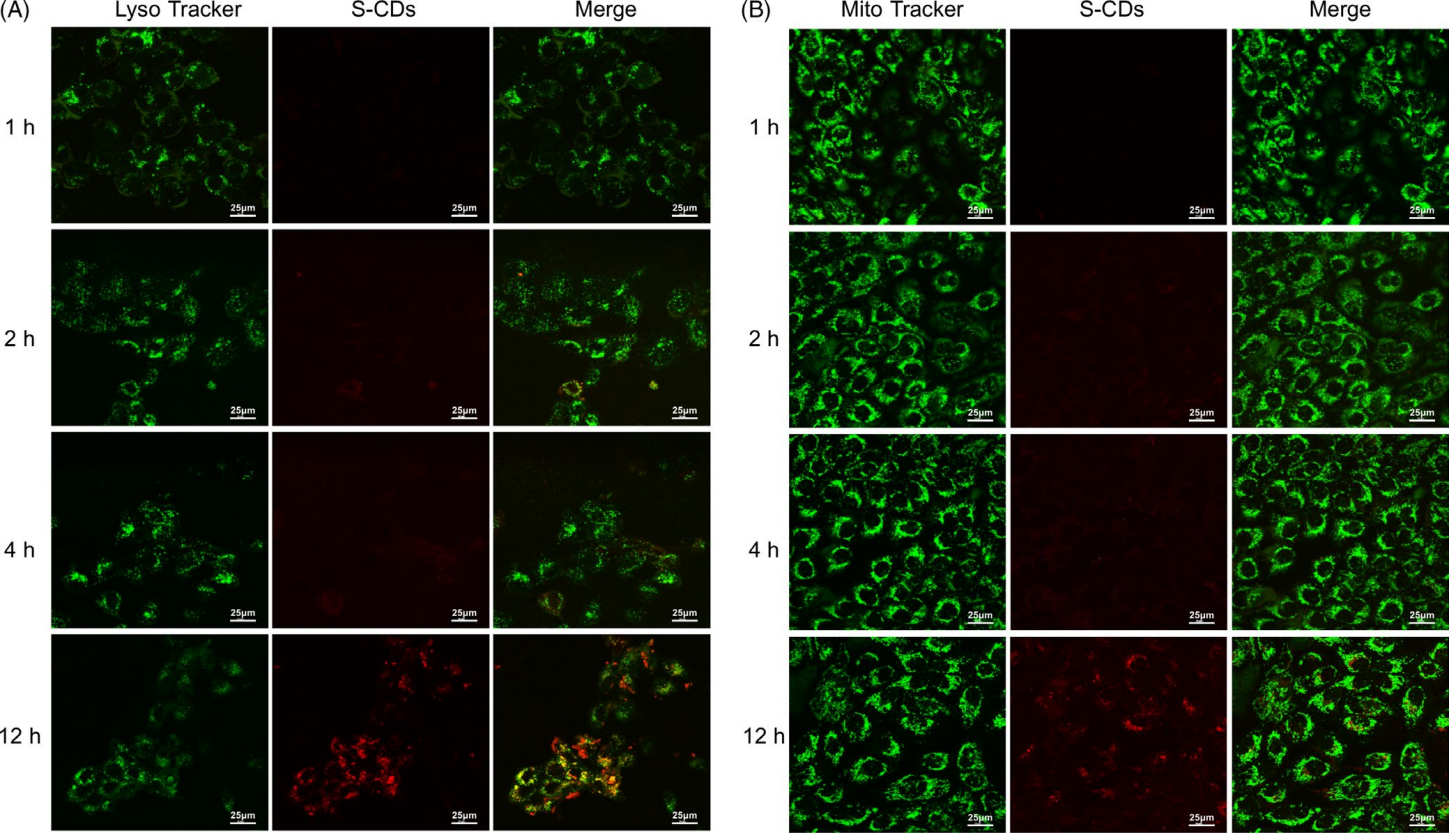
S‐CD major location—lysosome: (A) Confocal fluorescence images of UM1 cancer cells treated with S‐CDs (red), where the lysosome was shown in green (LysoTracker). (B) Confocal fluorescence images of UM1 cancer cells treated with S‐CDs (red), where the mitochondria were shown in green (MitoTracker). Scale bars are 25 μm All the measurements depicted were conducted in triplicate

**FIGURE A1:**
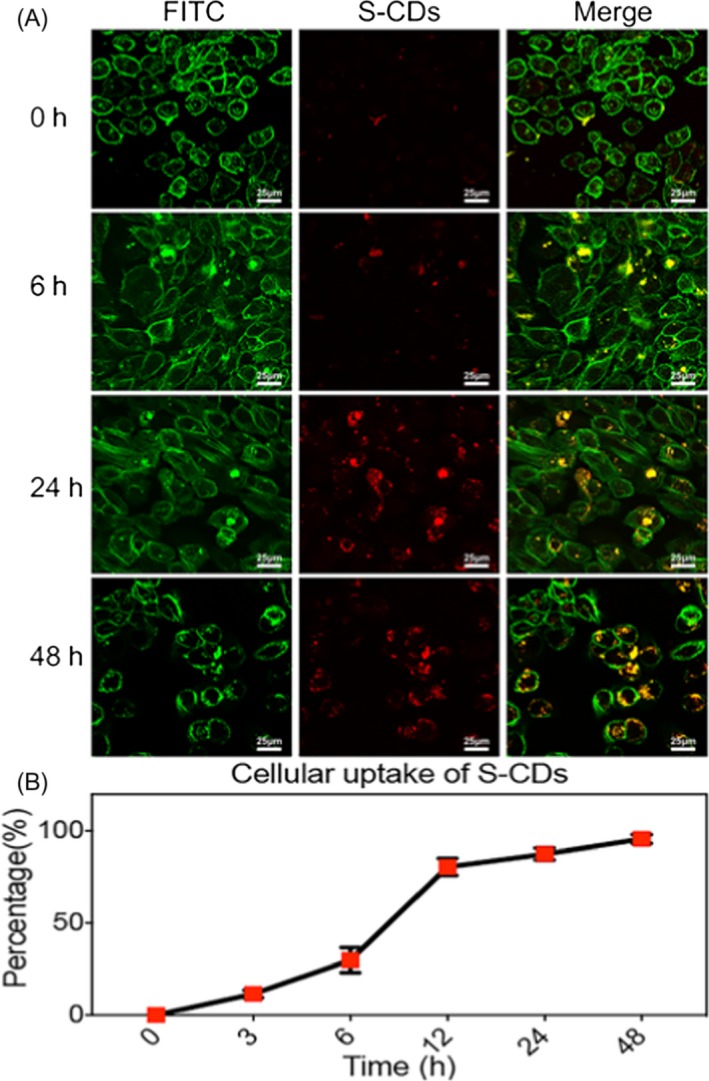
UM1 cellular uptake of S‐CDs. (A) Treated UM1 with S‐CDs for 0, 6, 24 and 48 h (1 µmol/L). Immunofluorescence images (S‐CDs: red, cytoskeleton: green). Scale bars are 25 μm. (B) Cellular uptake of S‐CDs by flow cytometry. Data are presented as mean ± SD (n = 3)

Previous reports^30^, ^31^ have verified that the curative effect of PDT primarily relied on the cellular uptake and localization of the photosensitizers. Considering the red fluorescence emission of the S‐CDs (∼600 nm). Therefore, the uptake of S‐CDs by UM1 cells was firstly characterized by using flow cytometry and confocal laser scanning microscopy. As shown in Figure A1A, during the first 24 hours of incubation, the red fluorescent signal of S‐CDs was significantly increased and then slightly increased for another 48 hours, which was consistent with the results from flow cytometer (Figure A1B). Besides, the positive charge on the surface of the S‐CDs yielded promising cell uptake. After 24 hours of incubation, the S‐CDs could be detected in about 90% of the UM1 cells. The confocal laser scanning microscopy suggested that the S‐CDs were accumulated in both lysosomes (Figure 3A) and mitochondria (Figure 3B) after incubating for 2 hours. Ten hours later, the fusion signal of red and green fluorescence in lysosomes was brighter, indicating that the S‐CDs mainly accumulated in lysosomes.

### 3.3 Promising PDT efficacy of the S‐CDs

**FIGURE 4:**
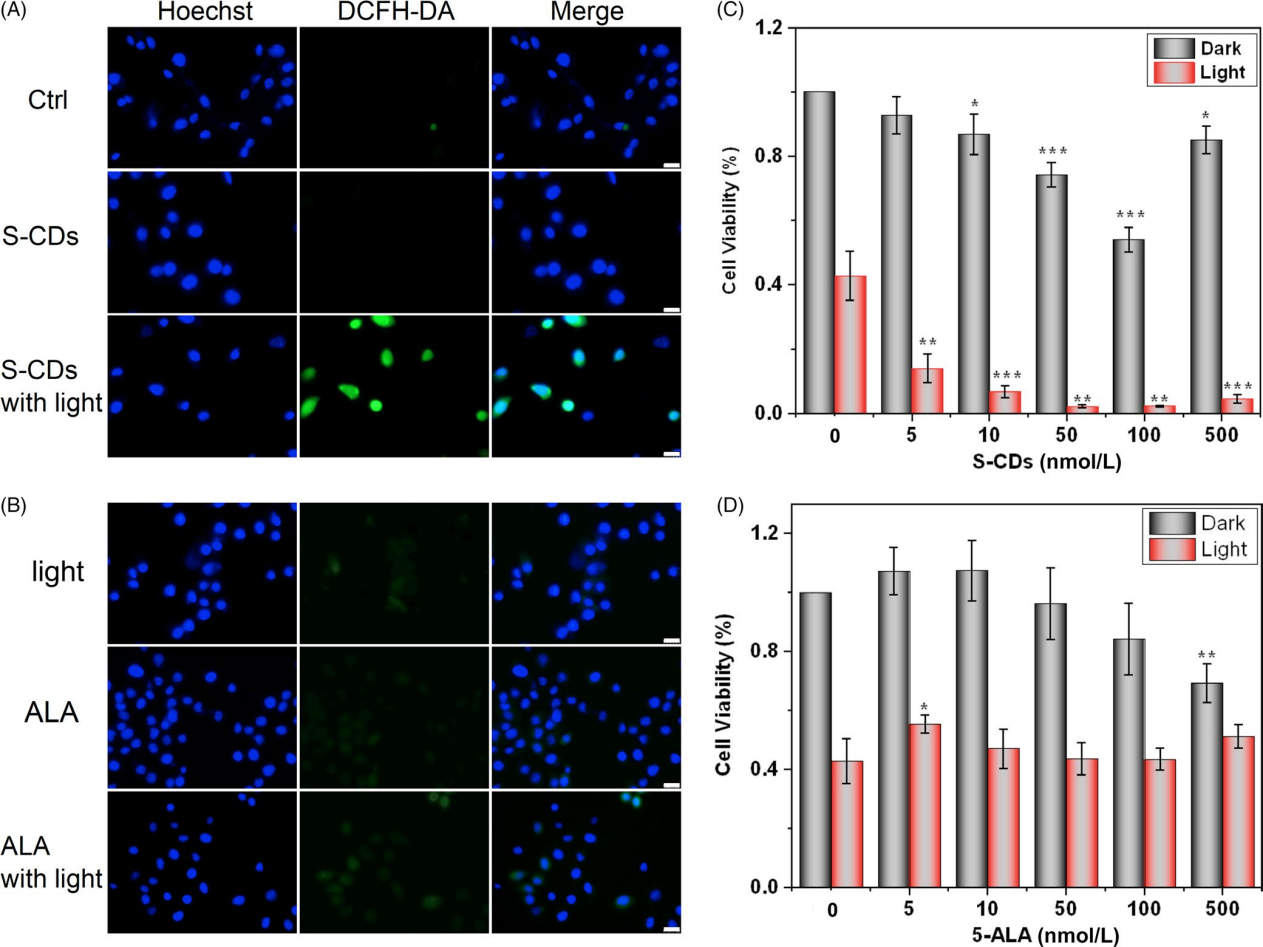
S‐CDs (40 nmol/L) have higher PDT efficiency than 5‐ALA (40 nmol/L): (A) Fluorescent images of ROS generated during S‐CD‐mediated PDT (scale bars are 25 μm). (B) Fluorescent images of ROS generated during 5‐ALA ‐mediated PDT (scale bars are 25 μm). (C) Cell Counting Kit‐8 to evaluate the cytotoxicity of different concentration of S‐CDs with and without illumination. (D) Cell Counting Kit‐8 to evaluate the cytotoxicity of different concentration of 5‐ALA with and without illumination. All the measurements depicted were conducted in triplicate. Data are presented as mean ± SD (n = 3). Statistical analysis: *P < .05. **P < .01. ***P < .001

**FIGURE A2:**
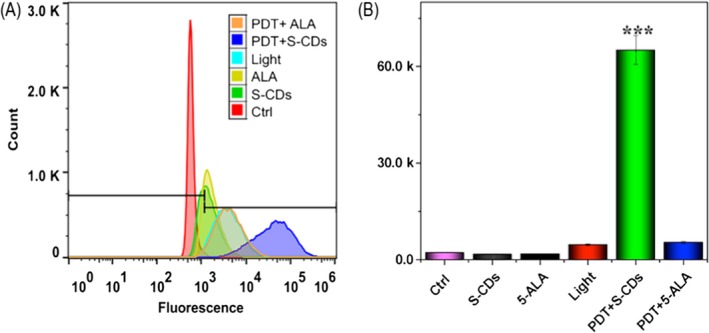
In vitro PDT performance of S‐CDs (40 nmol/L): (A) Flow cytometry chart of ROS generated during S‐CD‐mediated PDT. (B) Bar chart of ROS generated during S‐CD‐mediated PDT

In PDT, ROS could directly or indirectly damage cellular constituents through reacting with biological molecules, which plays a vital role in inducing cell apoptosis.^32^ Thus, the production of ROS that triggered by S‐CD‐mediated PDT was examined with the DCFH‐DA fluorescence assay. Bright green fluorescence was observed in the UM1 cells receiving S‐CD‐mediated PDT, while no obvious green fluorescent signal was discovered in the groups receiving S‐CDs or 5‐ALA without light irradiation as well as the group that merely received culture medium (Figure 4A). Among the UM1 cells that were administrated with 5‐ALA with light irradiation, only a faint green fluorescence can be detected (Figure 4B). The results demonstrated that the capability to generate singlet oxygen of the S‐CDs was higher than that of the 5‐ALA, and the S‐CD‐mediated PDT had the highest singlet oxygen yield, which was further confirmed by the flow cytometry results. As shown in the Figure A2, the purple solid line represented the group receiving the S‐CDs in the presence of light, which shifted significantly to the right compared with the other five groups. The bar chart presented the statistical analysis of the flow cytometry result and showed that the group receiving the S‐CDs in the presence of light (green bar) had the highest average fluorescence intensity generated by ROS among the six groups, which was in line with the former flow cytometry results.

Moreover, CCK‐8 assay was applied to determine the cytotoxicity and PDT efficiency when the S‐CDs was applied to perform PDT against UM1 cells. As illustrated in Figure 4C, a decrease in cell viability was observed in the cells receiving S‐CDs of 10 nmol/L‐100 nmol/L without the presence of light (grey bars), suggesting a low cytotoxicity. However, this trend changed when the concentration reached 500 nmol/L. The cell receiving 5‐ALA without light irradiation demonstrated similar cell viability down‐regulation at the concentration of 500 nmol/L (Figure 4D), suggesting low cytotoxicity. The viability of the UM1 cells receiving no effective agent with the presence of illumination was significantly dropped (Figure 4C,D, red bar, first column), which indicated the blue light itself could have certain effects on UM1 viability. Under illumination, as soon as cellular uptake of the S‐CDs occurred, the cell viability was immediately decreased, and the trend was consistent along with the increasing of S‐CD concentration (5 nmol/L, 20%, 100 nmol/L, 5%) (Figure 4C), while the 5‐ALA group presented similar cell viability as the illumination‐only group (Figure 4D).

**FIGURE A3:**
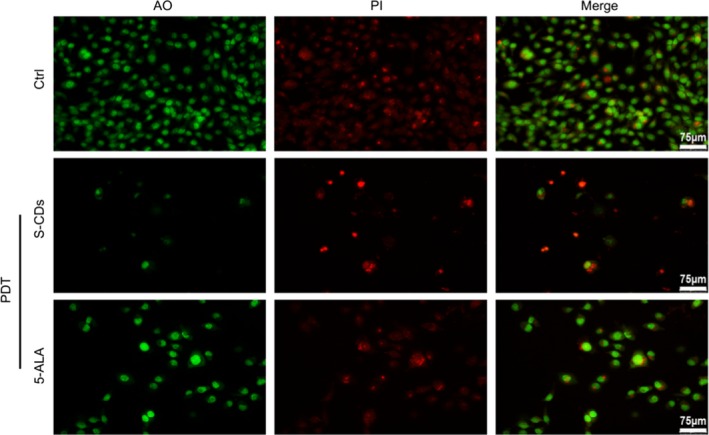
The (AO/PI) fluorescent intensity changes after PDT medicate S‐CDs (40 nmol/L) treatments. Immunofluorescence images (AO: green, PI: red). Scale bars are 75 μm

Besides, AO/PI assay was used to further evaluate the high photosensitive oxidation capacity of the S‐CDs. The nucleic‐acid‐highly‐affinitive AO penetrated membrane, causing a green fluorescent signals. While the PI only stained the DNA and the RNA of dead/dying cells with red fluorescence. As shown in Figure A3, the S‐CD group with light exposure experienced cell morphology change from spindle‐like (control group) into round, and some of them even scattered into fragments forming red luminous points of varied sizes. In the 5‐ALA group with light exposure, only slightly scattered red fluorescence could be detected and no drastic appearance change was observed in cell morphology, which was coincided with the CCK‐8 results.

### 3.4 S‐CD mediated cell apoptosis

**FIGURE A4:**
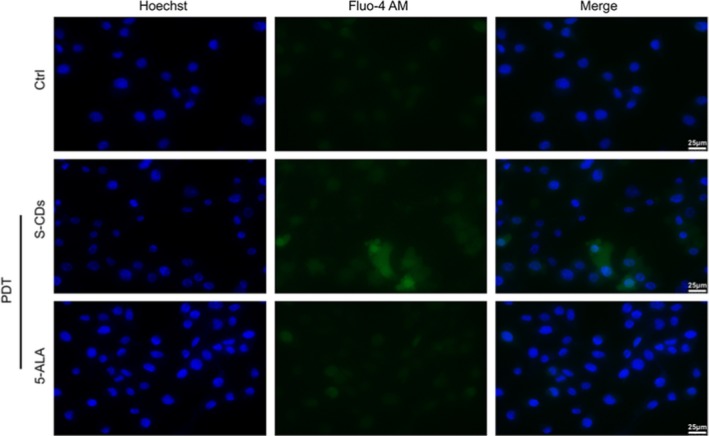
The Fluo‐4 AM fluorescent intensity changes after PDT + S‐CDs (40 nmol/L) treatments. Immunofluorescence images (Hoechst: blue, Fluo‐4 AM: green). Scale bars are 25 μm

To further emphasize the perfect PDT performance of S‐CDs for oral squamous cell carcinoma, the detailed intracellular responses to S‐CD‐mediated PDT were discussed. Firstly, as PDT treatment could raise the concentration of calcium (Ca^2+^) in cytoplasm, resulting in cell death,^33^, ^34^ the alteration in Ca^2+^ level during light exposure was evaluated by using Fluo‐4 AM (a Ca^2+^ probe). As illustrated in Figure A4, the green fluorescence in S‐CD‐treated group with light irradiation was more remarkably than the control groups, while only a faint green fluorescent signal was detected in the group receiving 5‐ALA‐mediated PDT, indicating larger amount of Ca^2+^ entered into cells after the S‐CD mediated PDT treatment. While for 5‐ALA‐mediated PDT, only a faint green fluorescence was visible.

**FIGURE 5:**
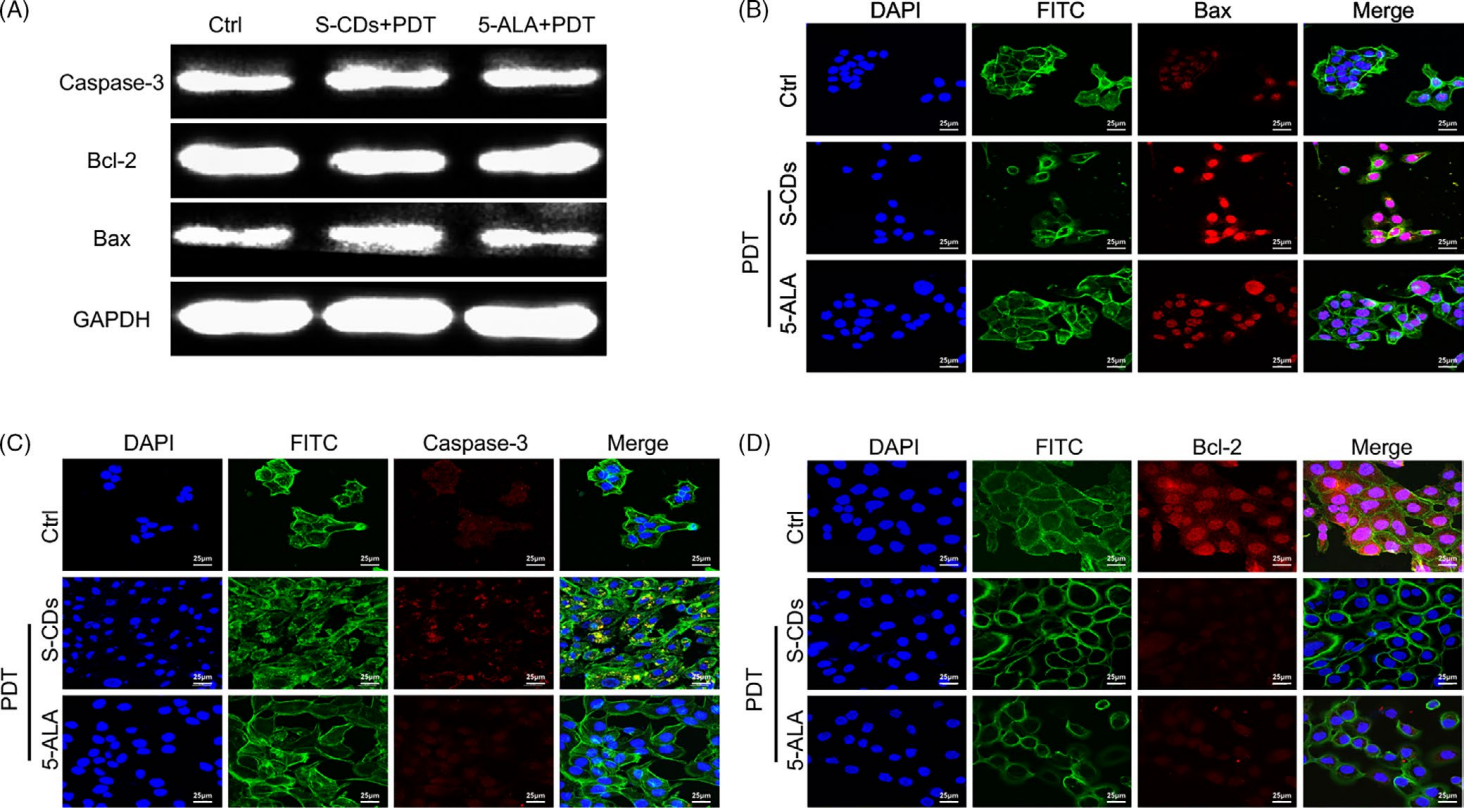
S‐CDs involved photodynamic therapy lead to cell apoptosis: (A) Expression of Bcl‐2, Bax and caspase‐3 determined by Western blotting. (B, C, D) After exposure with visible light and S‐CDs and 5‐ALA (40 nmol/L), immunofluorescent images of UM1 cells (Bcl‐2, Bax and caspase‐3: red, nucleus: blue, cytoskeleton: green). Scale bars are 25 μm

In addition, studies have shown that ROS induced cell apoptosis through mitochondrial apoptotic pathway, causing the change of Bcl‐2 family proteins (such as Bcl‐2 and Bax), followed by activation of caspase.^35^, ^36^, ^37^ To further trace the high therapeutic efficiency of S‐CDs at biomolecular level, Western blots and immunofluorescence analysis were employed to investigate the Bcl‐2, Bax and caspase‐3 (apoptosis‐related protein) after S‐CD‐mediated PDT. As shown in Figure 5A, the Western blot demonstrated a decreased relative fluorescence intensity of Bcl‐2 in the group receiving S‐CD‐mediated PDT. Meanwhile, reversed trends were observed in terms of the Bax and caspase‐3 proteins.

The confocal laser microscopy image revealed similar observation. In the group receiving S‐CD‐mediated PDT, the fluorophore intensity of the Bcl‐2 was the faintest among the three study proteins (Figure 5D), while the fluorescent signals of Bax and caspase‐3 were significantly stronger than those of the control group (Figure 5B,C). With respect to 5‐ALA at the same concentration, the fluorescent intensities of Bax and caspase‐3 were weaker, but the signal of Bcl‐2 was stronger than that of the S‐CD group. The results further confirmed the high PDT efficiency of S‐CDs.

## 4 DISCUSSION

In our study, the novel sulphur‐doped carbon dots (S‐CDs) were synthesized as a nano‐photosensitizer to concur the limitations of traditional photosensitizers in curing oral cancer, including undesirable singlet oxygen (^1^O_2_) quantum yields and long‐term phototoxicity. The well‐dispersed S‐CDs possessed a broad absorption (360‐600 nm) companied with red light emission, which was in favour of the application in real‐time cell imaging and photodynamic therapy for superficial malignant diseases. From the perspective of in vitro studies, the flow cytometry and laser confocal microscopy results showed that 90% of the UM1 cells could uptake the S‐CDs after 24 hours of incubation, and it was speculated that the positive charge on the surface of the S‐CDs greatly contributed to cell uptake, lying foundation for the initiation of further therapy.

Besides, the in vitro PDT simulating experiments were performed by using CCK‐8 and ROS probe (DCFH‐DA). The higher killing rate and reactive oxygen species production in the group receiving S‐CD‐mediated PDT indicated that the S‐CDs possessed higher photo‐oxidative activity than 5‐ALA at the same concentration. The yield of singlet oxygen was also significantly improved, which could be due to the effect of heavy atom doping. Besides, the study of cellular organelle localization of the S‐CDs showed that the nano‐photosensitizer initially entered into both lysosomes and mitochondria, and then mainly accumulated in lysosomes. Meanwhile, the higher singlet oxygen yield of the S‐CD nanoparticles in mitochondria and lysosome may contribute to the associated release amount of calcium (Ca^2+^) from internal stores to cytoplasm in the group receiving S‐CD‐mediated PDT, ulteriorly initiating cell apoptosis.

To further trace the therapeutic efficiency of the S‐CDs at biomolecular level, the apoptosis‐associated‐proteins—Bcl‐2, Bax and caspase‐3 as well as Ca^2+^, were investigated after the administration of S‐CD‐mediated PDT by using immune fluorescent staining and Western blot analysis. Firstly, the Fluo‐4 AM analysis indicated that the green fluorescent signal of the S‐CD‐mediated PDT group was brighter than that of the 5‐ALA‐mediated PDT group, suggesting the high photosensitive oxidation capacity of nano‐photosensitizer. Secondly, among the UM1 cell receiving S‐CD PDT treatment, the Bcl‐2 protein level was obviously decreased, while the level of Bax was significantly up‐regulated. Therefore, we inferred that the minimum dosage S‐CDs provoked apoptosis of UM1 cells via up‐regulating the caspase‐3 and Bax and decreasing the Bcl‐2 level during the PDT. However, controlled investigations with 5‐ALA at the same concentration induced minimal activation of the mitochondria apoptosis pathway. We expect this material could be used in bioimaging, targeted drug delivery and photodynamic therapy.

In conclusion, the novel S‐CDs were synthesized as a nano‐photosensitizer for oral squamous cell carcinoma therapy, which was proved to have satisfactory cell internalization ability and self‐luminous, and depicted low cytotoxicity without the presence of light irradiation. Under the condition of light irradiation, the S‐CDs may act as a more effective nano‐weapon for anticancer therapy compared with traditional PS 5‐ALA. The high therapeutic efficiency of the nano‐structure was speculated to be realized by generating high rate of ^1^O_2_, inducing acute stress response and Ca^2+^ influx, and thereafter the overexpression of caspase‐3 and Bax proteins as well as the down‐regulation of Bcl‐2 protein were triggered. Hence, the newly developed S‐CDs might be a promising alternative photosensitizer for the treatment of oral‐maxillofacial carcinoma by using PDT.

## CONFLICT OF INTEREST

The authors declare no potential conflicts of interest with respect to the authorship and/or publication of this article.

## AUTHOR CONTRIBUTIONS

Qirong Li contributed to conception, design, data acquisition and interpretation, performed all statistical analyses, drafted the manuscript and critically revised the manuscript. Ronghui Zhou synthesize S‐CDs, contributed to conception, design and interpretation, and critically revised the manuscript. Yu Xie draw the scheme picture and contributed to conception and design. Yanjing Li and Yu Chen contributed to conception and design and critically revised the manuscript. Xiaoxiao Cai contributed to conception and design, and critically revised the manuscript. All authors gave their final approval and agreed to be accountable for all aspects of the work.

## Data Availability

The data that support the findings of this study are available from the corresponding author upon reasonable request.

## References

[1] Alexiades‐Armenakas M . Laser‐mediated photodynamic therapy. Clin Dermatol. 2006;24:16‐25. 16427502 10.1016/j.clindermatol.2005.10.027

[2] Konopka K , Goslinski T . Photodynamic therapy in dentistry. J Dent Res. 2007;86:694‐707. 17652195 10.1177/154405910708600803

[3] Henderson BW , Dougherty TJ . How does photodynamic therapy work? Photochem Photobiol. 2010;55:145‐157. 10.1111/j.1751-1097.1992.tb04222.x 1603846

[4] Dolmans DEJGJ , Kadambi A , Hill JS , et al. Vascular accumulation of a novel photosensitizer, MV6401, causes selective thrombosis in tumor vessels after photodynamic therapy. Cancer Res. 2002;62:2151‐2156. 11929837

[5] Busch TM , Wileyto EP , Emanuele MJ , et al. Photodynamic therapy creates fluence rate‐dependent gradients in the intratumoral spatial distribution of oxygen. Cancer Res. 2002;62:7273‐7279. 12499269

[6] Gollnick SO , Liu X , Owczarczak B , Musser DA , Henderson BW . Altered expression of interleukin 6 and interleukin 10 as a result of photodynamic therapy in vivo. Cancer Res. 2013;57:3904‐3909. 9307269

[7] Peng Q , Berg K , Moan J , Kongshaug M , Nesland JM . 5‐Aminolevulinic acid‐based photodynamic therapy: principles and experimental research. Cancer. 2010;65:235‐251. 10.1111/j.1751-1097.1997.tb08549.x 9066303

[8] Liao JC , Roider J , Jay DG . Chromophore‐assisted laser inactivation of proteins is mediated by the photogeneration of free radicals. Proc Natl Acad Sci USA. 1994;91:2659‐2663. 8146171 10.1073/pnas.91.7.2659 PMC43429

[9] Bulina ME , Chudakov DM , Britanova OV , et al. A genetically encoded photosensitizer. Nat Biotechnol. 2005;24:95‐99. 16369538 10.1038/nbt1175

[10] Postiglione I , Barra F , Aloj SM , Palumbo G . Photodynamic therapy with 5‐aminolaevulinic acid and DNA damage: unravelling roles of p53 and ABCG2. Cell Prolif. 2016;49:523‐538. 27389299 10.1111/cpr.12274 PMC6496272

[11] Postiglione I , Chiaviello A , Aloj SM , Palumbo G . 5‐aminolaevulinic acid/photo‐dynamic therapy and gefitinib in non‐small cell lung cancer cell lines: a potential strategy to improve gefitinib therapeutic efficacy. Cell Prolif. 2013;46:382‐395. 23869760 10.1111/cpr.12040 PMC6622218

[12] Grant WE , Hopper C , Macrobert AJ , Speight PM , Bown SG . Photodynamic therapy of oral cancer: photosensitisation with systemic aminolaevulinic acid. Lancet. 1993;342:147‐148. 7687318 10.1016/0140-6736(93)91347-o

[13] Chen HM , Yu CH , Lin HP , et al. Successful treatment of an early invasive oral squamous cell carcinoma with topical 5‐aminolevulinic acid‐mediated photodynamic therapy. J Dent Sci. 2010;5:36‐40.

[14] Jui‐Chang T , Chun‐Pin C , Hsin‐Ming C , et al. Photodynamic therapy of oral dysplasia with topical 5‐aminolevulinic acid and light‐emitting diode array. Lasers Surg Med. 2010;34:18‐24. 10.1002/lsm.10250 14755421

[15] Shi S , Zhang L , Zhu M , et al. Reactive oxygen species‐responsive nanoparticles based on PEGlated Prodrug for targeted treatment of oral tongue squamous cell carcinoma by combining photodynamic therapy and chemotherapy. ACS Appl Mater Inter. 2018;10:29260‐29272. 10.1021/acsami.8b08269 30106279

[16] Josefsen L , Boyle R . Photodynamic therapy: novel third‐generation photosensitizers one step closer? Brit J Pharmacol. 2010;154:1‐3. 10.1038/bjp.2008.98 PMC2438986 18362894

[17] Lismont M , Dreesen L , Wuttke S . Metal‐organic framework nanoparticles in photodynamic therapy: current status and perspectives. Adv Funct Mater. 2017;27:1606314.

[18] Liu Z , Chen K , Davis C , et al. Drug delivery with carbon nanotubes for in vivo cancer treatment. Cancer Res. 2008;68:6652‐6660. 18701489 10.1158/0008-5472.CAN-08-1468 PMC2562710

[19] Zhang X , Meng L , Lu Q , Fei Z , Dyson PJ . Targeted delivery and controlled release of doxorubicin to cancer cells using modified single wall carbon nanotubes. Biomaterials. 2009;30:6041‐6047. 19643474 10.1016/j.biomaterials.2009.07.025

[20] Li G , Zhou T , Lin S , Shi S , Lin Y . Nanomaterials for craniofacial and dental tissue engineering. J Dent Res. 2017;96:725‐732. 28463533 10.1177/0022034517706678

[21] Zheng XT , Ananthanarayanan A , Luo KQ , Chen P . Glowing graphene quantum dots and carbon dots: properties, syntheses, and biological applications. Small. 2015;11:1620‐1636. 25521301 10.1002/smll.201402648

[22] Hola K , Zhang Y , Wang Y , Giannelis EP , Zboril R , Rogach AL . Carbon dots—Emerging light emitters for bioimaging, cancer therapy and optoelectronics. Nano Today. 2014;9:590‐603.

[23] Martinsjúnior PA , Alcântara CE , Resende RR , Ferreira AJ . Carbon nanotubes: directions and perspectives in oral regenerative medicine. J Dent Res. 2013;92:575‐583. 23677650 10.1177/0022034513490957

[24] Ge J , Lan M , Zhou B , et al. A graphene quantum dot photodynamic therapy agent with high singlet oxygen generation. Nat Commun. 2014;5:4596. 25105845 10.1038/ncomms5596 PMC4143951

[25] Nakayama S , Sasaki A , Mese H , Alcalde RE , Matsumura T . Establishment of high and low metastasis cell lines derived from a human tongue squamous cell carcinoma. Invasion Metastasis. 2000;18:219‐228. 10.1159/000024515 10729767

[26] Nakayama S , Sasaki A , Mese H , Alcalde RE , Tsuji T , Matsumura T . The E‐cadherin gene is silenced by CpG methylation in human oral squamous cell carcinomas. Int J Cancer. 2001;93:667‐673. 11477576 10.1002/ijc.1386

[27] Hsieh HJ , Cheng CC , Wu ST , Chiu JJ , Wung BS , Wang DL . Increase of reactive oxygen species (ROS) in endothelial cells by shear flow and involvement of ROS in shear‐induced c‐fos expression. J Cell Physiol. 2015;175:156‐162. 10.1002/(SICI)1097-4652(199805)175:2<156::AID-JCP5>3.0.CO;2-N 9525474

[28] Li H , Hu H , Zhao Y , et al. Multifunctional aptamer‐silver conjugates as theragnostic agents for specific cancer cell therapy and fluorescence‐enhanced cell imaging. Anal Chem. 2015;87:3736‐3745. 25686206 10.1021/ac504230j

[29] Ljubojevic S , Radulovic S , Sedej S , Kockskaemper J , Pieske B . Early alterations of nuclear Ca2+‐dependent signalling in cardiac hypertrophy. Eur Heart J. 2013;34:32‐32.

[30] Zhang JX , Zhou JW , Chan CF , et al. Comparative studies of the cellular uptake, subcellular localization, and cytotoxic and phototoxic antitumor properties of ruthenium(II)–porphyrin conjugates with different linkers. Bioconjugate Chem. 2012;23:1623‐1638. 10.1021/bc300201h 22770381

[31] Xu H , Ma S , Liu Q , et al. A naphthalimide‐aminal‐based pH‐sensitive fluorescent donor for lysosome‐targeted formaldehyde release and fluorescence turn‐on readout. Chem Commun (Camb). 2019;55:7053‐7056. 31143885 10.1039/c9cc02481f

[32] Simon HU , Haj‐Yehia A , Levi‐Schaffer F . Role of reactive oxygen species (ROS) in apoptosis induction. Apoptosis. 2000;5:415‐418. 11256882 10.1023/a:1009616228304

[33] Granville DJ , Ruehlmann DO , Choy JC , et al. Bcl‐2 increases emptying of endoplasmic reticulum Ca2+ stores during photodynamic therapy‐induced apoptosis. Cell Calcium. 2001;30:343‐350. 11733941 10.1054/ceca.2001.0243

[34] Ruck A , Heckelsmiller K , Kaufmann R , Grossman N , Haseroth E , Akgun N . Light‐induced apoptosis involves a defined sequence of cytoplasmic and nuclear calcium release in AlPcS4‐photosensitized rat bladder RR 1022 epithelial cells. Photochem Photobiol. 2000;72:210‐216. 10946575 10.1562/0031-8655(2000)072<0210:LIAIAD>2.0.CO;2

[35] Lawen A . Apoptosis—an introduction. BioEssays. 2003;25:888‐896. 12938178 10.1002/bies.10329

[36] Liu X , Kim CN , Yang J , Jemmerson R , Wang X . Induction of apoptotic program in cell‐free extracts: requirement for dATP and cytochrome c. Cell. 1996;86:147‐157. 8689682 10.1016/s0092-8674(00)80085-9

[37] Zou H , Li Y , Liu X , Wang X . An APAF‐1· cytochrome c multimeric complex is a functional apoptosome that activates procaspase‐9. J Biol Chem. 1999;274:11549‐11556. 10206961 10.1074/jbc.274.17.11549

